# Water-Related Parasitic Diseases in China

**DOI:** 10.3390/ijerph10051977

**Published:** 2013-05-17

**Authors:** Shan Lv, Li-Guang Tian, Qin Liu, Men-Bao Qian, Qing Fu, Peter Steinmann, Jia-Xu Chen, Guo-Jing Yang, Kun Yang, Xiao-Nong Zhou

**Affiliations:** 1 National Institute of Parasitic Diseases, Chinese Center for Disease Control and Prevention, Key Laboratory of Parasite and Vector Biology, Ministry of Health, Shanghai 200025, China; E-Mails: lvshan000@126.com (S.L.); jztlg@126.com (L.-G.T.); liuqin0901@sohu.com (Q.L.); ahtlqmb-007@163.com (M.-B.Q.); fuqing1981@yahoo.com.cn (Q.F.); peter.steinmann@unibas.ch (P.S.); chenjiaxu@yahoo.com (J.-X.C.); 2 WHO Collaborating Centre for Malaria, Schistosomiasis and Filariasis, Shanghai 200025, China; 3 Department of Epidemiology and Public Health, Swiss Tropical and Public Health Institute, University of Basel, Basel 4051, Switzerland; 4 Jiangsu Institute of Parasitic Diseases, Wuxi 214064, China; E-Mails: guojingyang@hotmail.com (G.-J.Y.); jipdyk@163.com (K.Y.); 5 School of Public Health and Primary Care, The Jockey Club Chinese University of Hong Kong, Shatin, Hong Kong

**Keywords:** parasitic disease, water, protozoa, helminths, epidemiology, China

## Abstract

Water-related parasitic diseases are directly dependent on water bodies for their spread or as a habitat for indispensable intermediate or final hosts. Along with socioeconomic development and improvement of sanitation, overall prevalence is declining in the China. However, the heterogeneity in economic development and the inequity of access to public services result in considerable burden due to parasitic diseases in certain areas and populations across the country. In this review, we demonstrated three aspects of ten major water-related parasitic diseases, *i.e.*, the biology and pathogenicity, epidemiology and recent advances in research in China. General measures for diseases control and special control strategies are summarized.

## 1. Introduction

Water is a precondition for life, including that of all parasites and other organisms that infect humans. Indeed, many infectious diseases are water-related, *i.e.*, they directly depend on water bodies for their spread and transmission or as a habitat for intermediate or final hosts [1]. 

Water-related parasites can be categorized into three groups according to their transmission route. The first group is associated with drinking water, which may be contaminated with cysts or oocysts, larvae, or eggs from various parasites. The second group is transmitted via penetration of the human skin during water contact. The parasites in this group may swim freely in water until they find a human host. The transmission of the third group of parasites depends on the consumption of uncooked freshwater products, e.g., plants, fish, snails or crustaceans. Obviously, the first two groups are closely related to water contact, while the key element of transmission of the third group is not water, but hosts and vectors living in the water.

This review focuses on the most important members of water-related parasitic diseases in China. Since their prevalence is influenced by the provision of clean water and sanitation, they are a priority of many rural development programmes [2]. We review will focus on the biology and pathogenicity, epidemiology and research advances of ten water-related parasitic diseases in China, and hope that this review will increase the recognition of these conditions in China and stimulate more studies in the area.

## 2. Methods

We reviewed the scientific literature on water-related parasitic diseases: amoebiasis, giardiasis, cryptosporidiosis, cyclosporiasis, blastocystosis, schistosomiasis, fascioliasis, fasciolopsiasis, clonorchiasis, and paragonimiasis. Considered publications were published from 1 January 1990 to 31 December 2011. The English language literature was obtained from ScienceDirect Onsite, SpringerLink and PubMed, while the Chinese language literature was obtained through CNKI (Chinese National Knowledge Infrastructure), Wanfang, and VIP.

For each disease, we firstly reviewed its biology and pathogenicity of that disease. Then, its epidemiology in China, particularly the two nationwide community-based sampling surveys on major human parasitic diseases which had been carried out during 1988–1992 (thereafter named “First National Survey”) and 2001–2004 (“Second National Survey”) respectively [3,4]. Thirdly, recent advances in research are introduced. We conclude with a discussion of control strategies for water-related diseases.

## 3. Results and Discussion

### 3.1. Amoebiasis

#### 3.1.1. Parasite and Pathogenicity

**Figure 1:**
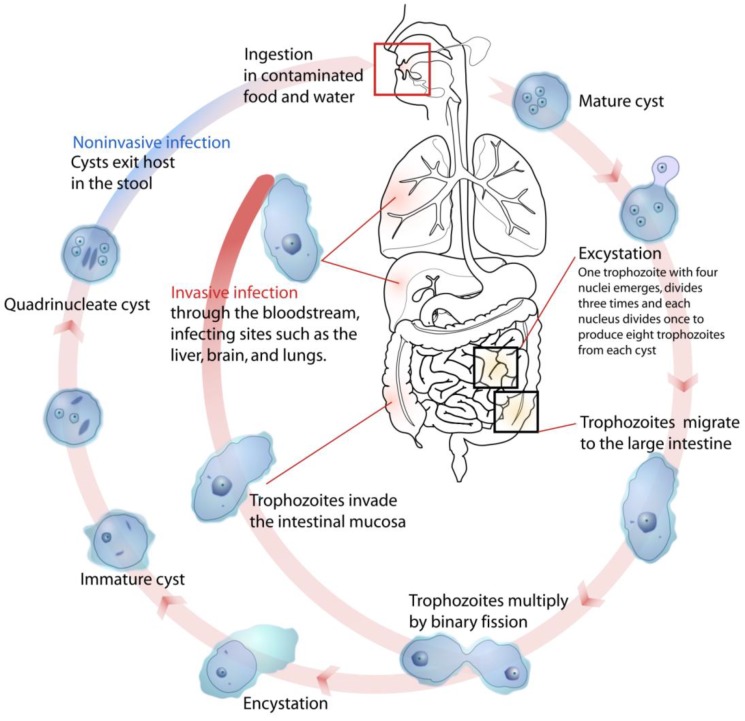
Life cycle of *Entamoeba histolytica* (Available online: http://en.wikipedia.org/wiki/File:Entamoeba_histolytica_life_cycle-en.svg).

Pathogenic amoebiasis is caused by the protozoan parasite *Entamoeba histolytica* (Figure 1). Globally, the parasite causes an estimated 100,000 deaths per year and is one of the most important parasitic infections, ranking third in terms of public health relevance after malaria and schistosomiasis [5]. *E. histolytica* had been recognized as early as 1875 [6] and associated with variable morbidity. In 1993, the morphologically identical [7] but non-pathogenic *E. dispar* was described as a separate species [8], explaining the absence of morbidity in many amoebiasis cases. The failure of microscopic examinations to distinguish between the two species complicates the diagnosis in resource-constrained settings where specific ELISA or PCR tests are often not available. 

Infection is by ingestion of cysts (generally from fecally contaminated food or water). Excystation occurs in the ileum of the small intestine. Trophozoites multiply by binary fission in the large intestine. Most trophozoites remain in the lumen of the intestine. Cyst formation is triggered by dehydration of gut contents. Invasive forms of the disease lead to amoebic dysentery in which the trophozoites invade the intestinal wall, leading to the formation of amoebic ulcers. This results in severe diarrhea with blood and mucus in stools. If trophozoites penetrate the intestinal wall, serious health problems can occur, including liver abcesses (the most common manifestation), or spread to the lungs and brain, usually resulting in death, or other organs or tissues (e.g. pleura, pericardium, genitor-urinary system).

*E. histolytica* is a major cause of dysentery. Four major intestinal syndromes include asymptomatic colonization, acute amoebic colitis, fulminant colitis and amoeboma [9] (Table 1). 

**Table 1:** Major syndromes of *E. histolytica* infection.

Syndrome	Symptoms
Asymptomatic colonization (most common)	✔ No symptoms
Acute amoebic colitis	✔ Lower abdominal pain ✔ Frequent bloody stools, over several weeks ✔ Fever
Fulminant colitis (occurs most often in children)	✔ Diffuse abdominal pain ✔ Profuse bloody diarrhea ✔ Fever ✔ Liver abscess ✔ Colonic perforation
Amoeboma (approximately 1% of patients)	✔ Asymptomatic lesion of colon wall ✔ Tender mass accompanied by dysentery

#### 3.1.2. Epidemiology

Amoebiasis is wide spread in rural China, as illustrated by the results of the first national survey [4] when more than 14,000 participants or 0.95% of the total were found to be infected, resulting in a the country-wide infection estimate of about 10 million individuals. *E. histolytica* has been reported from almost all provinces and the prevalence exceeded 1% in twelve provinces. Prevalence generally increased with age, while the steepest increase occurs from birth to the age of 14 years. The prevalence among farmers and shepherds was significantly higher compared to other groups and it has been suggested that different socio-economic factors synergistically impacted the prevalence. The results of the second national survey indicated that the prevalence of amoebiasis had generally declined. For example, the prevalence of *E. histolytica* had decreased from 1.50% to 0.14% in Zhejiang Province [10] and from 0.57% to 0.13% in Henan Province [11]. 

While the overall prevalence of amoebiasis in China is declining, some studies indicate that the proportion of diarrhea attributable to *E. histolytica* is increasing, particularly among paediatric outpatients [12,13]. Additionally, several outbreaks among migrant workers and children have been reported. In 2004, an epidemic occurred in a township of Dongguan city where about 54.4% of 263 inpatients with diarrhea were parasitologically confirmed as amoebiasis cases [14]. Of them, 84.6% were migrants and 79.7% were classified as paediatric patients. Poor living conditions of migrant workers and incomplete treatment resulting in chronic infections were described as the primary factors for the epidemic occurrence of amoebiasis in this area. Another outbreak in a township of Jiangshan city (Zhejiang Province) involved children from several kindergartens and primary schools [15]. 

Parasitic co-infections of HIV/AIDS patients are of particular concern [16]. In a cohort study involving participants from three provinces, the seroprevalence of *E. histolytica* among HIV-infected individuals was 12.1%, four times the rate among non-HIV-infected individuals (3.1%) [17]. 

#### 3.1.3. Recent Advances in Research

Research pertaining to *E. histolytica* had been initiated relatively early in China when compared to other intestinal protozoa. In addition to numerous clinical reports of intestinal amoebiasis, extra-intestinal abscesses due to ectopic location of *E. histolytica* and amoebiasis due to other species have been highlighted recently. The significance of *E. histolytica* for animal health is also drawing attention [18,19]. Since traditional microscopic diagnosis of *E. histolytica* with or without iodine staining routinely fails to differentiate *E. histolytica* from *E. dispar*, enzyme-linked immunosorbent assays (ELISA) for *E. histolytica* in stool samples have been developed and evaluated [15,20,21]. Promising polymerase chain reaction (PCR) tests for detecting parasite DNA directly in liver abscess aspirates or serum sample have also been developed [21]. However, PCR is rarely used in China for the diagnosis of the parasite in stool samples, although good results have been reported from other countries [22]. 

### 3.2. Giardiasis

#### 3.2.1. Parasite and Pathogenicity

**Figure 2:**
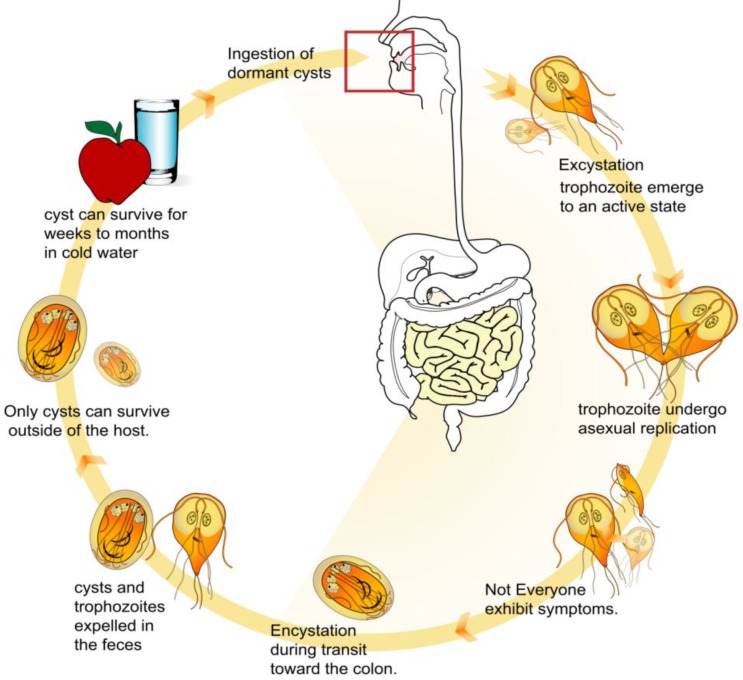
Life cycle of *Giardia intestinalis* (Available online: http://en.wikipedia.org/wiki/File:Giardia_life_cycle_en.svg).

Giardiasis is caused by *Giardia intestinalis* (also called *G. lamblia* or *G. duodenalis*) and is one of the most common causes of parasitic diarrhea. Today, giardiasis, along with cryptosporidiosis, continues to represent the major parasite-related public health concern of water utilities in developed nations [23]. *G.* *intestinalis* occurs worldwide and is a zoonotic parasite in certain areas while in others, the infection is believed to be limited to humans [24,25]. *G. intestinalis* exists in two forms, namely a trophozoite (the active form) and a cyst (the inactive form) (Figure 2).

The motile trophozoite has two nuclei, four pairs of flagella, and one or two curved median bodies of unknown function. Reproduction is by binary fission; no sexual process is known. The infective stage is an oval cyst, which is excreted in the faeces and ingested with contaminated food or water. The cyst contains four small nuclei, grouped at one end, and a confused jumble of flagella, median bodies *etc*. in the centre. 

Diarrhea and associated symptoms may occur in various forms, depending on the stage of infection (Table 2). Symptoms pertaining to ectopic parasitism can be observed during the chronic stage; cholecystitis [26,27,28] and pancreatitis [29] have been associated with *G. intestinalis* infections, and the parasite has been isolated from ascites, pleural effusions and joint fluids [30,31,32]. The mechanisms involved in fatal giardiasis cases are not clear [32,33].

**Table 2:** Stages and symptoms of Giardiasis.

Stage	Symptoms
Prodromal	✔ Intestinal uneasiness ✔ Nausea ✔ Anorexia
Acute (often lasts 3–4 days, subsiding spontaneously)	✔ Explosive, watery, foul-smelling diarrhea ✔ Low-grade fever ✔ Chills ✔ Abdominal bloating and cramping ✔ Vomiting ✔ Distention, associated with flatulence ✔ Blood and mucus in stools (rare)
Chronic	✔ Intermittent diarrhea ✔ Abdominal bloating and cramping ✔ Weight loss ✔ Malnutrition ✔ Growth retardation

#### 3.2.2. Epidemiology

**Figure 3:**
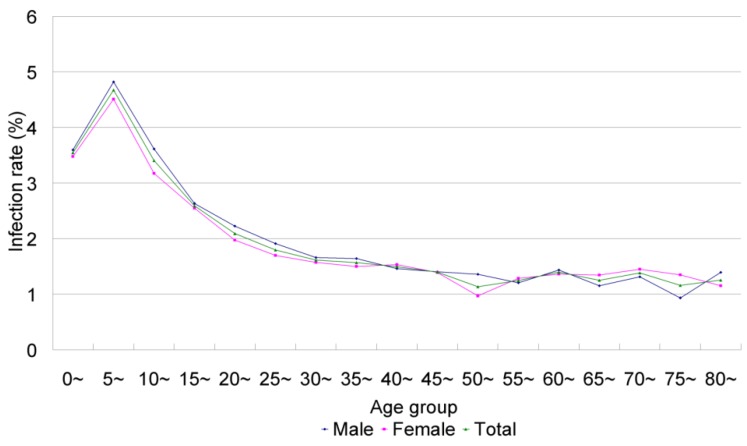
Change of infection rate of *Giardia intestinalis* prevalence by age and sex inChina; theoriginaldataareobtainedfromXu *et al*. [4].

Giardiasis is the best-known intestinal protozoan infection in China where many field surveys have been conducted to reveal its epidemiology. Giardiasis occurs across the country and the overall prevalence has been estimated at 2.52% following the first national survey [4], translating into 28.5 million infections. The highest prevalences were found in Xinjiang Uyghur (9.26%) and Tibet autonomous regions (8.22%) and Henan Province (7.18%). Regarding age, children under 15 years were most affected with a peak prevalence of 4.67% in the age group 5–10 years (Figure 3). There was no significant difference between prevalences in males and females but family clustering was observed. 

The prevalence of giardiasis in China is currently declining. According to the second national survey, the *G. intestinalis* prevalence had decreased significantly, e.g., from 7.18% in Henan Province and 3.85% in Zhejiang Province to 2.55% and 1.00%, respectively [10,11]. Interestingly, the distribution of *G. intestinalis* does not always follow that of intestinal helminth infections, probably reflecting differences in the way of transmission with *G. intestinalis* more dependent on water and soil-transmitted helminths more dependent on faecal contamination of the solid environment. However, regional idiosyncrasies are also important. The prevalence of *G. intestinalis* was only 1.9% in a population in south Yunnan Province where the prevalence of each of the common soil-transmitted helminth species exceeded 85%. Earlier observations had already noted that the prevalences of pathogenic intestinal protozoa were generally low in southeast Asia [34,35].

Although *G. intestinalis* infection is widespread in the general population, it is believed to be responsible for only a small fraction of all diarrhea cases. In a study conducted between 1996 and 2001, only 0.32% of 3,116 diarrheal patients were found to be infected with *G.* *intestinalis* while the combined prevalence of all intestinal prototoa was 21.7%. The majority of all intestinal protozoa were *E. histolytica*, infecting 17.65% of the samples [13]. Similarly, only 0.15% of 1,354 diarrheal patients were found to be infected with *G.* *intestinalis* in another study [36]. 

#### 3.2.3. Recent Advances in Research

Besides basic epidemiological surveys, ectopic and severe infections have recently received more attention. Infections in the joints [32], tonsil [37], gall bladder [27,38,39] and thoracic cavity [30] have been described, helping to understand the true pathogenicity of *G. intestinalis*. Studies on viral infections of *G.* *intestinalis* will facilitate the study of mechanisms for invasion [40,41]. 

*G.* *intestinalis* and *Cryptosporidium* spp. are the protozoan parasites most frequently found in water bodies [42,43,44]. With increasing awareness of the importance of safe water supply, quality criteria for drinking water and standard examination methods have been proposed and implemented in China since 2007. The detection methods are based on the method 1623 “*Cryptosporidium* and *Giardia* in water by filtration/IMS/FA” initially published by the U.S. Environmental Protection Agency but certain procedures have been modified. 

*G.* *intestinalis* has a zoonotic component and understanding its animal origins is crucial for the control of giardiasis. Based on electrophoretic evidence, there are at least seven valid assemblages (A–G) [45], of which humans can be infected by assemblages A and B. The morphological identification of assemblages is difficult, rendering genetic biomarkers the major tool recently. The triose phosphate isomerase and ITS-5.8SrDNA genes are considered the best markers. Genetic analysis also showed that isolates with different host origins or from several geographic locations might share the same gene type. Therefore, host species and geographic isolation may play a subordinate role in the genetic diversity of *G.* *intestinalis* [46,47].

Many protozoa have been found to be infected by a virus [48]. The *Giardia lamblia* virus (GLV) was first described as a specific double-stranded RNA virus in 1986 [49]. GLV isolated from humans in Beijing, China have been sequenced and appear to be identical to isolates from other places [41]. In another study performed in Changchun, the virus from *G. canis* was sequenced and found to be highly similar to GLV [50]. Based on GLV1515-2322, the coding part of the coat protein, an antiserum of GLV1518-2322 was prepared in order to detect GLV in *G.* *intestinalis* [51]. 

### 3.3. Cryptosporidiosis

#### 3.3.1. Parasite and Pathogenicity

**Figure 4:**
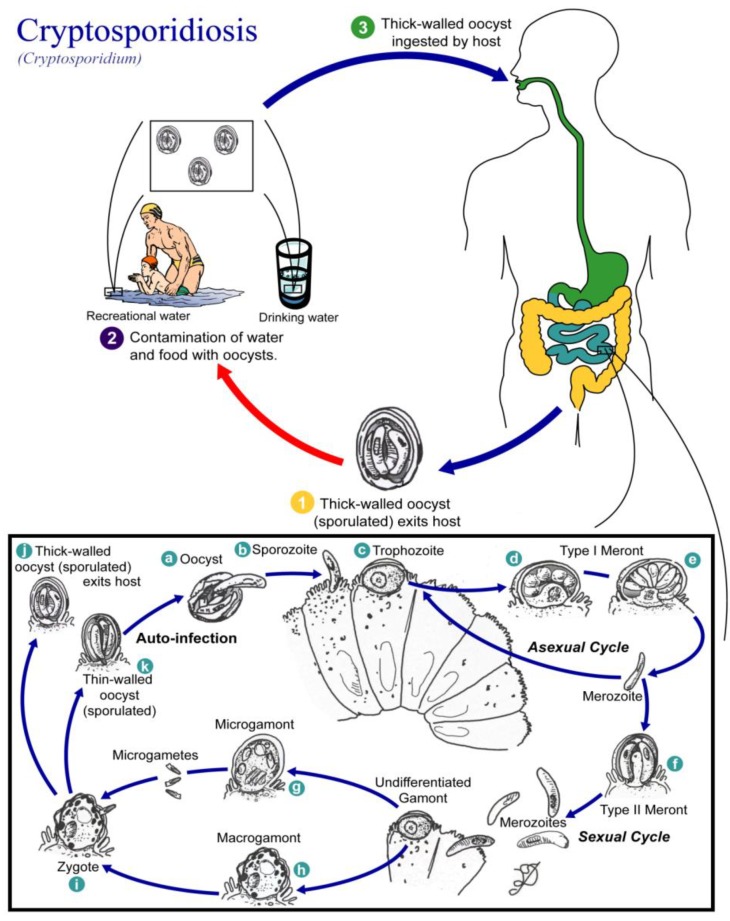
Life cycle of *Cryptosporidium* spp. (Available online: http://en.wikipedia.org/wiki/File:Cryptosporidiosis_01.png).

Cryptosporidiosis is a zoonosis caused by several *Cryptosporidium* species (Figure 4). The parasite was initially described from the gastric glands of laboratory mice and identified as a new species in 1912 [52]. 

The first cases of human cryptosporidiosis were independently reported from an immuno-compromised adult and a child in 1976 [53,54]. *Cryptosporidium* spp. are widely distributed in the environment. Many animals, including livestock and poultry, have been identified as sources of infection and the prevalence in animals is often considerably higher than that in humans [55,56,57].

*Cryptosporidium* spp. consists of an asexual stage and a sexual stage. After being ingested, the oocysts excyst in the small intestine. They release sporozoites that attach to the microvilli of the epithelial cells of the small intestine. From there they become trophozoites that reproduce asexually by multiple fission, a process known as schizogony. The trophozoites develop into Type I meronts that contain eight daughter cells. These daughter cells are Type 1 merozoites, which get released by the meronts. These merozoites can cause autoinfection by attaching to epithelial cells. They can aslo become Type II meronts, which contain 4 Type II merozoites. These merozoites get released and attach to the epithelial cells. From there they become either macrogamonts or microgamonts, the female and male sexual forms, respectively. This stage, when sexual forms arise, is called gametogony. Zygotes are formed by microgametes from the microgamont penetrating the macrogamonts. The zygotes develop into oocysts of two types. 20% of the oocysts have thin walls and so can reinfect the host by rupturing and releasing sporozoites that start the process over again. The thick-walled oocysts are excreted into the environment. The oocysts are mature and infective upon being excreted. They can survive in the environment for months.

The incubation period in humans is about 5 to 28 days [52]. Infections with *C. parvum* have a wide range of manifestations, from asymptomatic to life-threatening disease (Table 3). Although *C.* *parvum* usually resides in the small intestine, symptomatic cryptosporidiosis has also been found to involve other digestive-tract organs, the lungs and possibly the conjunctiva [58].

**Table 3:** Effects of cryptosporidiosis related to prior physiological state of patient.

Patient Health	Effects
Immunocompetent	✔ No effect ✔ Cholera-like diarrhea; may contain mucus, but seldom blood and leukocytes ✔ Abdominal pain ✔ Nausea and vomiting ✔ Fever ✔ Spontaneous recovery after short illness
Immunocompromised	✔ Above symptoms, leading to dehydration and weight loss ✔ Prolonged and potentially fatal illness

#### 3.3.2. Epidemiology

Cryptosporidiosis is an increasing concern in the face of climbing HIV infection numbers in China. The first cryptosporidiosis case in China was reported in 1987 from Nanjing, Jiangsu Province [59]. At least 938 confirmed cases had been reported by 1998, more than 86.6% of them paediatric cases [4]. Table 4, Table 5 summarize the results of major *C. parvum* surveys among patients with diarrhea and the general population in China. An estimated 1.4%–10.4% of all diarrhea episodes can be attributed to *Cryptosporidium* (Table 4). Prevalences among the general population range from 0.79% to 6.59% (Table 5). An important reason for apparent variations in local prevalence are the epidemic nature of the disease and the use of different diagnostic techniques [60,61,62]. The prevalence varies between different places but no significant decrease following socioeconomic development can be discerned.

Children, particularly those under 5 years old, are more vulnerable to *Cryptosporidium* spp. infection than older age groups and the prevalence among pre-school age children is higher both among patients with diarrhea and in the general population. Generally, the prevalence is not significant different between males and females but prevalence is higher in rural and suburban areas compared to urban ones.

The pathogenicity of *C. parvum* is known to vary depending on the immune status. However, few investigations have specifically targeted immunosuppressed populations, such as patients with HIV/AIDS or cancer, in China. A recent survey showed that 4.25% (9/212) of the screened AIDS patients were infected with *C.* *parvum* [63], with a higher prevalence among those who refused antiretroviral treatment (21.21%; 7/33). Cancer and chemo-radiotherapy often impair the immune system and thus promote *Cryptosporidium* infections. Two thirds of 108 patients with cancer were confirmed to be infected with *Cryptosporidium* in a recent study, and chemotherapy tended to be associated with higher prevalence than radiotherapy or combination therapy [64]. Several studies have also demonstrated a high prevalence among illegal drug addicts. In a study conducted in Dali, Yunnan Province, 16.8% (84/500) of all injection drug users (IDUs) were found to be infected with this parasite upon stool examination while the prevalence was 6% among the general population [65]. In a similar study conducted in a drug rehabilitation center in Changsha, Hunan Province, the prevalence was 19.05% [66]. Of note, serological testing may result in higher prevalence estimates; in a study in Nanjing, Jiangsu Province a total of 69.9% of 588 IDUs were seropositive for specific antibodies as compared to 29.4% (113/384) among the general population, a significant difference [67].

**Table 4:** The prevalence of cryptosporidiosis in patients with diarrhea in China.

Report time	Location (City, Province)	No. exam	No. infected	Infection rate	Sex		Age		Reference
					male	female	Children	Adults	
1992	Yuxi, Yunnan	1,640	84	5.12	-	-	7.99(52/651) (<5 years); 5.00(7/140) (6–10 years); 3.95(9/228) (11–20 years)	2.41(11/457) (21–60years); 4.81(5/104) (>60 years)	Fan *et al*, [68]
1993	Kaifeng, Henan	483	12	2.48	-	-	2.48(<4 years)		Su *et al*. [69]
1998	Harbin, Heilongjiang	931	13	1.40	-	-	1.40(<4 years)		Zhao *et al*. [70]
1999	Wenzhou, Zhejiang	1,060	60	5.66	5.18	6.38	6.13(59/962) (<15 years)	1.02(1/98) (>15 years)	Xing *et al*. [71]
2000	Hangzhou, Zhejiang	548	57	10.40	10.31	10.60	10.40(<10 years)		Lu *et al*. [72]
2001	Chengdu, Sichuan	406	12	2.96	-	-	2.96(<10 years)		Zhang *et al*. [73]
2001	Gansu Province	1,840	41	2.23	2.01	2.50	10.95(23/210) (<6 years); 1.82(7/385) (7–17 years)	0.88(11/1245) (>18 years)	Chen *et al*. [74]
2002	Urumqi, Xinjiang	190	4	2.11	0.86	4.05	1.56(1/64) (1–4 years.); 0.00(0/34) (5–11 years);	0.00(0/12) (>60 years)	Luo *et al*. [75]
2002	Yunnan Province	378	20	5.29	5.11	5.45	7.09(9/127) (<15 years)	4.44(10/225) (15–60 years); 3.85(1/26) (>60 years)	Zhang *et al*. [76]
2003	Huainan, Anhui	827	46	5.56	5.23	6.06	6.12(45/735) (<5 years)	1.09(1/92) (>15 years)	Cai *et al*. [77]
2004	Longhai, Fujian	248	7	2.82	1.48	5.95	3.75(3/80) (<4 year)		Xu *et al*. [78]
2006	Qiqihar, Heilongjiang	330	11	3.33	2.69	4.17	5.61(6/107) (<15 years)	1.98(4/202) (15–60 years); 4.76(1/21) (>60 years)	Niu *et al*. [79]
2006	Heze, Shandong	237	6	2.53	2.00	3.40	3.85(3/78) (<1 year); 2.17(2/92) (<5 years); 0.00(0/11) (5–18 years);	1.79 (1/56) (18–72 years)	Zhou [80]
2006	Shenyang, Liaoning	283	9	3.18	3.95	2.30	6.98(6/86) (<18 years)	1.52 (3/198) (>18 years)	Li *et al*. [81]

**Table 5:** The prevalence of cryptosporidiosis in the general population in China.

Report time	Location (City, Province)	No. exam	No. Infected	Infection rate	Sex		Age		Reference
					male	female	Children	Adult	
1991	Nanjing, Jangsu	2,018	16	0.79	-	-	0.79(<5 years)		Shen *et al*. [82]
1991	Xuzhou and Huaiyin, Jiangsu	2,613	67	2.56	-	-	3.29(59/1794)	0.98(8/819)	Xu *et al*. [83]
1992	Qingdao, Shandong	969	26	2.68	2.86	2.47	2.68(preschool and primary school)		Gong *et al*. [84]
1992	Hunan Province	3,739	69	1.85	1.86	1.83	2.52(57/2262) (<10 years); 1.99(10/503) (10–20 years)	0.21(2/974) (>20 years.)	Lu *et al*. [85]
1992	Yuxi, Yunnan	2,853	42	1.47	1.32	1.60	2.66(17/640) (<10 years); 1.47(10/680) (10–20 years)	0.92(12/1308) (21–60 years); 1.33(3/225) (>60 years)	Fan *et al*, [68]
1993	Jiangsu Province	5,089	89	1.75	1.91	1.54	3.18(57/1793) (<4 years); 0.97(32/3296) (4–15 years)		Chen *et al*. [86]
1995	Xinjiang Province	1,124	53	4.72	32	21	10.59(34/321) (<5 years); 2.37(19/803) (6–10 years)		Wang *et al*. [87]
2001	Weifang, Shandong	1,943	55	2.83	2.73	2.97	2.83(5–13 years)		Cui *et al*. [88]
2004	Anhui Province	1,204	42	3.49	3.59	3.37	3.45(preschool children)		Lu *et al*. [62]
2006	Qiannan, Guizhou	1,739	40	2.30	2.23	2.39	8.76(12/137) (<2 years); 3.76(7/186) (3–6 years); 1.99(4/201) (7–12 years); 2.77(8/289) (13–17 years)	0.97(9/926) (>18 years)	Wang *et al*. [89]
2007	Shiyan, Hubei	941	62	6.59	6.88	6.26	6.59 (7–16 years.)		Zhu *et al*. [90]
2007	Shiyan, Hubei	1,118	51	4.56	4.60	4.52	4.56 (3–6 years.)		Zhu *et al*. [91]
2009	Shiyan, Hubei	2,549	119	4.67	4.82	4.51	5.99(110/1836)	1.26(9/713)	Zhu *et al*. [92]
2009	Nanjing, Jiangsu	1,758	17	0.97	0.94	1.00	0.97		Du *et al*. [61]

#### 3.3.3. Recent Advances in Research

The importance of *Cryptosporidium* in China is increasingly recognized. A high number of epidemiological investigations both in humans and animals have been conducted in the whole country, and prevalences in different population segments have been mapped. Co-infections of HIV and *Cryptosporidium* have attracted particular attention [93,94]. *Cryptosporidium* has been isolated from a range of animals and the taxonomy is still debated. Consequently, more and more studies are looking for suitable biomarkers and identification techniques to differentiate isolates [95]. Genetic variations have also been exploited for the development of source-tracing techniques [96]. Isolation techniques and diagnostic methods are also being developed in China. PCR-based techniques are the main methods currently applied to detect *Cryptosporidium* [97]. 

### 3.4. Cyclosporiasis

#### 3.4.1. Parasite and Pathogenicity

Human cyclosporiasis is caused by *Cyclospora cayetanensis* and has been identified as an important cause of diarrhea worldwide. *C. cayetanensis* was classified into the subphylum Apicomplexa, family Eimeriidae in 1993 [98] and received its current name in 1994 [99]. Humans are the only known host of this parasite and are infected when ingesting oocysts in contaminated water, food or soil. The role of animals in the transmission of *C. cayetanensis* is uncertain but of increasing concern.

Infections with *C. cayetanensis* are often transient but chronic infections have also been described (Table 6). The shedding of oocysts need not concur with symptoms. Although symptoms and oocyst excretion typically subside within a few days to 1 or 2 weeks, some untreated persons excrete oocysts for 11 month after symptoms resolve [100,101]. Persistence of symptoms for several weeks longer than oocyst excretion has also been documented [102]. 

**Table 6:** Stages and Symptoms of Cyclosporiasis.

Stage	Symptoms
Prodromal	✔ Flu-like symptoms
Acute (often lasts a few days to 1 or 2 weeks)	✔ Severe diarrhea ✔ Symptoms associated with gastroenteritis emerge with acute or gradual onset of watery diarrhea, anorexia, fatigue, and weight loss ✔ Loss of appetite, nausea, vomiting, abdominal bloating and cramps, body aches, fever and chills, headache, and constipation ✔ Ascending infection of biliary tract, in patients with AIDS
Chronic	✔ Extended duration of symptoms above ✔ Fatigue ✔ Malaise

#### 3.4.2. Epidemiology

Since 1995 when the first confirmed case of cyclosporiasis was reported in Fujian Province, China [103], *C. cayetanensis* has become an increasing concern in patients with diarrhea. The prevalence varies widely between places with higher prevalences usually found in tropical and humid areas at low elevation [104]. The prevalence in rural populations is higher than in urban populations [105,106]. 

Although *C. cayetanensis* is transmitted via similar routes as *Cryptosporidium* spp., their presence may be asymmetrical. For example, in a survey performed in Xishan County, Yunnan Province, *C. parvum* was diagnosed in 13% of all patients with diarrhea, while none of them was found to be infected with *C. cayetanensis* [104]. Many studies demonstrate paediatric patients with diarrhea are more likely to be infected with *Cryptosporidium* spp., whereas a recent study showed that the prevalence of cyclosporiasis in the group above 60 years was significantly higher than in younger age groups [106]. The proportion of *C. cayetanensis* infections may be particularly elevated in chronic diarrhea patients. For example, a study showed that 8.2% of all patients with chronic diarrhea were infected with *C. cayetanensis* while only 2.5% of the patients with acute diarrhea were infected with this parasite [106]. *C. cayetanensis* infections also impact the immune status. A study revealed that cyclosporiasis patients had a decreased level of CD3^+^ and CD4^+^, while CD8^+^ was normal [107]. 

#### 3.4.3. Recent Advances in Research

Although the prevalence of cyclosporiasis among diarrheal patients can be considerable as documented in several provinces of China, few epidemiological surveys have been implemented and many techniques applied to cryptosporiasis have not been introduced to cyclosporiasis research. Consequently, one challenge in epidemiological surveys is the lack of sensitive techniques to differentiate *C. cayetanensis* from other intestinal protozoa. Indeed, misdiagnosis is common [108]. 

Humans currently are the only known host of *C. cayetanensis*. The lack of an animal model prevents studying this parasite [109]. To overcome this limitation, Ge and colleagues established a rat model for *C. cayetanensis*. They first suppressed the immune system of rats using hydrocortisone or cyclophosphamide and then infected them with *C. cayetanensis*. The number of oocysts in stool gradually increased and reached a peak 5–7 days after infection [110]. 

The susceptibility to *C. cayetanensis* infection is thought to be related to the status of the immune system. A series of relevant studies was performed in order to reveal the relationship between the likelihood of an infection and different immune status parameters [107,111,112]. It was found that among the infected, the titer of membrane interleukin-2 receptor, CD3^+^ and CD4^+^ was significantly decreased relative to non-infected individuals, while that of soluble interleukin-2 receptor as well as specific IgG and IgM were significantly elevated. 

### 3.5. Blastocystosis

#### 3.5.1. Parasite and Pathogenicity

*Blastocystis hominis* is one of the most frequently diagnosed protozoan parasites in human faecal samples. It is found in both symptomatic and healthy individuals and therefore, its pathogenic potential is still debated [113]. Additionally, many aspects of this organism including its taxonomy, life cycle and mode of transmission continue to be controversial despite this parasite being first discovered in human faeces as early as 1912 [114]. The morphologic diversity of the organism and the low sensitivity of the generally used wet-mount detection technique add further difficulties to its study [113].

Most people carrying *B. hominis* infections are asymptomatic while some show gastrointestinal symptoms including diarrhea, abdominal discomfort, abdominal pain or abdominal cramping and vomiting. Acute infections may cause watery diarrhea. In addition, fatigue, loss of appetite, bloating, and other non-specific gastrointestinal symptoms have been associated with *B. hominis* infections. 

#### 3.5.2. Epidemiology

*B. hominis* is endemic across the World, with a focus in tropical and subtropical regions. The first national survey showed that the overall prevalence *B. hominis* in China was 1.3% [4]. The parasite was found in 22 provinces; the highest provincial-level prevalence of *B. hominis* was 8.0% in Sichuan Province [4]. However, these figures probably underestimate the real situation. A recent study on *B. hominis* not only revealed that the prevalence can be as high as 32.6% in a subtropical rural area located in Yunnan Province and 1.9% in the urban population in Shanghai, but also showed that sensitive culture methods detect over 1.5 times more cases than a more conventional ether concentration approach [35,115]. 

The *B. hominis* prevalence in patients with diarrhea is considerable albeit it is often not clear whether their condition is due to the infection with this parasite. A recent study performed in Guangxi Zhuang Autonomous Region collected stool samples from 1,354 diarrheal outpatients among whom 18.5% were infected with *B. hominis* [36]. The proportion can be even higher in patients with chronic diarrhea [116]. Co-infection with other parasites is common; in the study from Guangxi mentioned above, 31.9% of all patients with *B. hominis* were simultaneously infected with other parasites [36]. The most common accompanying parasite, *Clonorchis sinensis,* accounted for 71.3% of all co-infections. Another survey showed an even higher co-prevalence of 51.1% among patients with *B. hominis* and one third of these co-infections were attributable to *G. intestinalis* [116]. Unlike cryptosporidiosis and cyclosporiasis, several studies found that middle-aged individuals are most susceptible to *B. hominis* [36,117]. 

**Figure 5:**
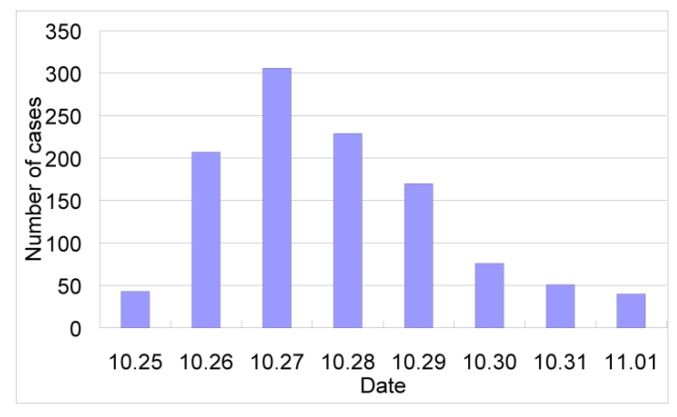
An outbreak of *Blastocystis hominis* in Hengshui Township, China. The original data are obtained from Wu *et al*. [118].

Diarrhea outbreaks have rarely been attributed to *B. hominis*. A big diarrhea outbreak presumably due to this parasite occurred in a township of Chongyi County, Jiangxi Province in 1996 [118] where 1,122 diarrheal patients were reported within eight days (Figure 5). Contaminated drinking water has been identified as the most likely source of infection (Table 7). 

**Table 7:** An outbreak of *Blastocystis hominis* in Hengshui Township, China.

School	No. students	Source of drinking water	No. cases	Infection rate (%)
Chongyi middle school	1,524	running water	106	6.96
Technical school	1,074	spring water	5	0.47
Hengshui middle school	608	well water	2	0.33
Chengguan primary school	2,326	running water	264	11.35
Chengguan kindergarten	411	running water	117	28.47
Woman united kindergarten	324	running water	62	19.14
Total	6,267		556	8.87

#### 3.5.3. Recent Advances in Research

Recent studies relevant to *B. hominis* have focused on diagnostics, the relationship between the parasite and diarrhea, and molecular epidemiology. Traditional methods to detect this protozoan parasite from stool sample include direct smears and staining with different dyes. A study compared three culture methods with different media, *i.e.*, RPMI1640, 199 and LES, and found RPMI1640 to be the most sensitive of them. In this medium, *B. hominis* can survive for a longer time and the final quantity of cells was the largest [119]. Recently, *in vitro* broth culture was confirmed to effectively improve the detection rate of *B. hominis* in human stool [120].

The pathogenicity of *B. hominis* has been confirmed in mice [121]. A case control study revealed that *B. hominis* infections in humans cause inflammation in the left colon and rectum [122]. The degree of impairment was positively correlated with the infection intensity. The titers of IL28, IL218 and GM2CSF in the intestinal mucous membrane were significantly elevated compared to healthy individuals, and again positively correlated with infection intensity. It has thus been concluded that *B. hominis* infections induce and mediate immune responses of epithelial cells that eventually result in inflammation.

A total of five genotypes including one which was previously unknown were found in a molecular epidemiological study in China [115]. The consumption of raw water plants was positively associated with subtype 1, and drinking unboiled water was positively associated with subtype 3. Genotype proportions varied between counties and mixed infections were common. It has been concluded that human infections with different genotypes might result from infections over different transmission routes and sources of infection [123]. 

### 3.6. Schistosomiasis

#### 3.6.1. Parasite and Pathogenicity

*Schistosoma japonicum*, the Asian or oriental schistosome, is endemic in China, the Phillipines and a small area of Indonesia. The morbidity due to schistosomiasis can mainly be attributed to the eggs trapped or dispersed on their way from the blood vessel-dwelling flukes to the intestine and the resulting immune reactions. The flukes themselves cause little disturbance [124]. Infection takes place in water bodies where the cercariae—larvae that emerge from intermediate host snails—actively target suitable end hosts and penetrate their skin. Schistosomiasis japonica has unique characteristics compared to the four other specise of human schistosomes, namely *S. masoni*, *S. hamatobium*, *S. intercalatum* and *S. mekongi*. Firstly, over 40 animal species, including cattle, pigs, dogs, cats and goats can serve as natural definitive hosts, rendering it a zoonotic rather than anthroponotic parasite. Secondly, the intermediate host snail *Oncomelania hupensis* is amphibious rather than aquatic. Third, the disease is characterized by more severe morbidity than that due to other species since the number of eggs produced per female worm is higher. Symptoms can become severe due to high infection intensities or extended exposure due to untreated illness (Table 8). 

**Table 8:** Stages and Symptoms of Schistosomiasis japonica.

Stages	Symptoms
Acute (exposure to high numbers of cercaria)	✔ High fever ✔ Hepatomegaly
Chronic (untreated acute infection)	✔ Liver fibrosis ✔ Liver cirrhosis ✔ Liver portal hypertension ✔ Splenomegaly ✔ Ascites ✔ Impaired physical and cognitive development
Infection outside intestines, liver and spleen	✔ Morbidity due to immune reactions to eggs trapped or dispersed in lungs, nervous system, and other organs

#### 3.6.2. Epidemiology

Schistosomiasis japonica is one of the most important parasitic diseases in China and its epidemiology has been extensively studied and reviewed [125]. When the national control programme was launched in the 1950s, the disease was common in many areas south of the Yangtze River, including Anhui, Fujian, Guangdong, Hubei, Hunan, Jiangsu, Jiangxi, Sichuan, Yunnan and Zhejiang Provinces, the Guangxi Zhuang Autonomous Region and Shanghai Municipality. The lowest-lying endemic area was recorded at sea level and the highest one in Yunnan Province at 3,000 m. The most severely affected areas were located along the Yangtze River, in the areas of the great lakes (*i.e.*, Dongting and Poyang lake) and surrounding areas [126]. Today, the parasite has been eliminated from Fujian, Guangdong, Guangxi, Zhejinag and Shanghai [125]. Substantial progress in controlling schistosomiasis has also been achieved in most of the remaining endemic areas and the total number of infected people has been reduced by over 90% since 1950 [127,128]. A nationwide sampling survey conducted in 2004 provided a detailed picture of the contemporary epidemiological situation and put the number of infections at 720,000 [129]. In 2008, the number of infections was estimated at about 412,000 [130].

The schistosome-endemic areas of China have been stratified into three types, based on ecosystem characteristics, namely the plain and water-network region, marshland and lake region, and hilly and mountainous region. The parasite has been eliminated from the formerly most heavily endemic plain and water-network region and has largely been brought under control in the hilly and mountainous regions. However, control in the marshland and lake regions proved difficult and the disease remains prone to resurgence [130].

#### 3.6.3. Recent Advances in Research

The successes with schistosomiasis control in China have been attributed to strong government support and sustainable yet adaptable policies [131]. Since the prevalence in human has declined significantly, animals, most notably livestock, have become the major source of infection in most areas. A recent study showed that a comprehensive control strategy focusing on interventions to reduce the transmission from cattle and humans to snails was highly effective [132]. This strategy has now been implemented in more than 90 counties across five endemic provinces and it has been suggested that the official target of reducing the prevalence in all endemic counties to less than 1% by 2015 could be achieved [132].

Disability adjusted life years (DALYs) have been widely used to measure disease burden and direct the allocation of health resources [133]. However, recent studies and meta-analyses challenge the current estimates of schistosomiasis-related diseases burden [134]. A recent study performed in China found that the overall disability weight of chronic schistosomiasis japonica was 0.191, with age-specific weights ranging from 0.095 to 0.246 [135]. These numbers are significantly higher than those used to evaluate global disease burden in 2004, namely 0.005 among those aged <15 years and 0.006 among those aged >15 years [136].

The transmission of *S. japonicum* is subject to environmental conditions and changes therein, including climate change [137,138,139]. It has been predicted that the potential endemic area will markedly expand northward and include an additional 783,883 km^2^ by 2050, accounting for 8.1% of the surface area of China [137]. Based on this prediction, efforts for adaptation and surveillance are ongoing [140]. 

The genomes of *S. japonicum* has been sequenced and published [141,142]. Transcriptomics and proteomics studies are in progress [143]. Vaccine development, drug discovery and improvements of diagnostic techniques are further research topics [144,145,146]. The mitochondrial genome of *O. hupensis* has also been studied, and genetic markers have been used to analyze the spatial distribution of the snail in China [147,148].

### 3.7. Fascioliasis

#### 3.7.1. Parasites and Pathogenicity

Human fascioliasis is caused by *F. hepatica* and *F. gigantica*. Both species are endemic in China. The life cycle of these two species are essentially similar [149]. They use herbivorous mammals as definitive host and freshwater snails (Lymnaeidae) as intermediate hosts [150]. The adult worms parasitize dwell in the biliary system of definitive hosts (cattle, sheep, *etc*.). The eggs are released to the intestine and exit the host body with faeces. Hatched miracidia invade freshwater snail host and develop to cercaria. The latter emerge from snails and stick to aquatic plants and finally become metacercariae. Humans and mammals acquire infection by ingesting aquatic plants contaminated with metacercariae [150]. 

Larval migration in the liver induces inflammation and hepatic dysfunction, which is the main base for acute symptoms (Table 9). Intermittent high fever and hepatalgia are the major symptoms at the acute stage [151,152,153,154]. The eosinophil count normally increases. The discovery of eggs in faeces is rare at this stage. Chronic symptoms coincide with the persistence of *Fasciola* spp. worms in the bile ducts. Many cases of chronic fascioliasis are asymptomatic. Jaundice appears when biliary obstruction occurs. Ectopic parasitism in cutaneous tissue, lung and other organs has also been reported [155,156]. Severe infections can lead to fatal outcome [154,157].

**Table 9:** Stages and symptoms of Fascioliasis.

Stages	Symptoms
Acute (last a few months)	✔ Fever (almost all patients) ✔ Hepatalgia (almost all patients) ✔ Hepatomegaly ✔ Tenderness in epigastrium ✔ Percussion pain in liver ✔ Weigh loss ✔ Anaemia
Chronic	✔ Jaundice ✔ Hepatalgia
Infection outside biliary system	✔ Cutaneous mass ✔ Symptoms related to involvement of lungs, peritoneum, brain, thyroid, epididymis, and eye

#### 3.7.2. Epidemiology

Fascioliasis is a common parasitic disease in domestic animals in China and hence causes huge economic losses. Human fascioliasis has rarely been reported in the past decades. *F. hepatica* is considered the major cause of animal and human fascioliasis in China. Before 1990 a total of 45 human cases had been reported, including one case of fascioliasis gigantica [157]. The first national survey discovered 148 fascioliasis hepatica and nine fascioliasis gigantica cases among 1.5 million participants [4]. The cases were distributed in 18 out of 31 provinces in China [4], which indicated a large number of people were at risk of fascioliasis. Compared to fascioliasis hepatica, all nine human cases caused by *F. gigantica* were localized in Hainan Island, the southernmost province of China. However, recent phylogenetic analysis of isolates from different areas in the country showed three distinct clades, *i.e.*, *F. hepatica*, *F. gigantica*, and the “intermediate” type [158]. *F. gigantica* is also distributed in southwest part of China, including Guangxi and Guizhou Provinces [159,160]. Therefore, the cases due to *F. gigantica* probably occurred in southwest China. 

Although the second national survey on major parasitic diseases did not include fascioliasis [3], sporadic reports of human cases impliy a low prevalence in the past two decades. A total of 49 human cases, excluding the cases identified in the first national survey, were recorded. Notably, 40 cases were reported in the last ten years, which indicates a growing prevalence of fascioliasis in China. 

#### 3.7.3. Recent Advances in Research

Fascioliasis is an important disease in livestock and hence veterinary studies are predominant in literature. In contrast, only few human case reports have been published in literature in recent years. Triclabendazole is the most efficacious drug for treating human fascioliasis. However, the drug is only registered in a few countries, and not in China [161]. The majority of all patients in China are treated with praziquantel. The distribution of triclabendazole should be promoted. 

In the last ten years, molecular identification of *Fasciola* species was performed. Mitochondrial gene (*cox*1 and *nad*1) and internal transcribed spacers (ITS1 and ITS2) revealed *F. hepatica*, *F. gigantica* and the intermediate form coexist in China [158,159,160,162,163]. However, the studies could not establish a clear geographic distribution of *Fasciola* spp. due to small sample size. 

### 3.8. Fasciolopsiasis

#### 3.8.1. Parasite and Pathogenicity

**Figure 6:**
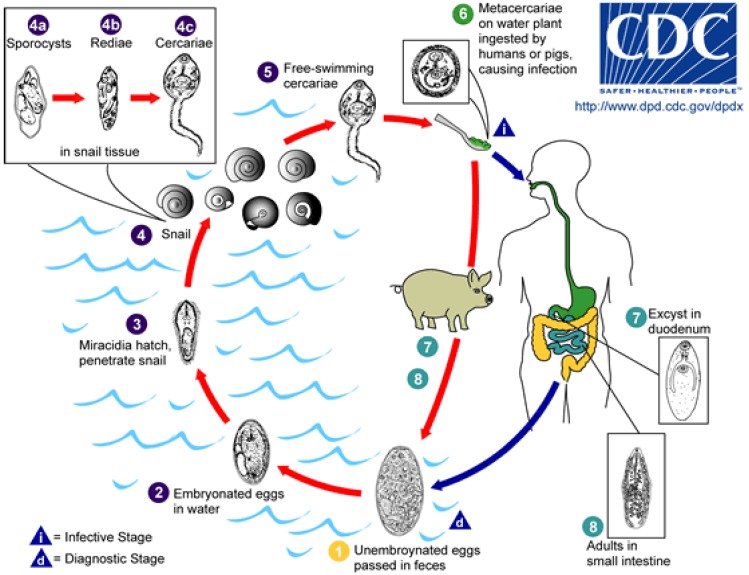
Life cycle of *Fasciolopsis buski* (Available online: http://commons.wikimedia.org/wiki/File:Fasciolopsis_buski_LifeCycle.gif).

Human fasciolopsiasis results from consuming water plants with cysts from *Fasciolopsis buski*, the largest intestinal fluke parasitizing humans (Figure 6). Encystation was also observed on the surface of aquarium wall, stones and other objects [164]. Cysts can also float on the water surface. Therefore, drinking fresh water may be an alternative route of transmission of *F. buski* [164].

Adults reside in the small intestine, and Immature eggs are released in stool. Eggs become embryonated in water and release miracidia, which invade a suitable snail intermediate host. In the snail the parasites undergo several developmental stages (sporocysts, rediae, and cercariae). The cercariae are released from the snail and encyst as metacercariae on aquatic plants. The mammalian hosts become infected by ingesting metacercariae on the aquatic plants. After ingestion, the metacercariae excyst in the duodenum and attach to the intestinal wall. There they develop into adult flukes in approximately 3 months, attached to the intestinal wall of the mammalian hosts (humans and pigs). The adults have a life span of about one year.

The symptoms of fasciolopsiasis manifest after an incubation period of 1–3 months and depend on several factors including the worm burden and physiologic state of the patient (Table 10).

**Table 10:** Symptoms of Fasciolopsiasis dependent on worm burden.

Worm Burden	Symptoms
Mild infection	✔ Abdominal pain ✔ Diarrhea ✔ Vomiting
Heavy infection	✔ Abdominal obstruction ✔ Obstructive jaundice, if worms ectopically lodge in the biliary system
Prolonged infection	✔ Malnutrition symptoms: weight loss, anemia, edema, *etc*. ✔ Stunting and mental retardation in children

#### 3.8.2. Epidemiology

Many reports indicate that fasciolopsiasis was heavily endemic in southern China in the 1970s and 1980s. For example, a survey performed in 8 villages in Putian County, Fujian Province, showed that 55.1% of 1,834 participants were infected with *F. buski* [165]. In the same area, 41.9% of the pig population and 35.6% of the examined water chestnut samples were infected. In another study, the prevalence was 12.1% among 61,392 individuals in Jiangling County, Hubei Province [166]. The first national survey reported 2,353 cases across China, a national prevalence of 0.17% [4]. It was extrapolated that there were 1.91 million infections with this parasite in 17 provinces. Above-average prevalences were found in Hubei, Hunan, Jiangsu, Gansu and Jiangxi Provinces as well as in Shanghai; Hubei had the highest prevalence of 1.89% and the prevalence was highest in the age group 50-54 years [4]. *F. buski* prevalence did not differ between sex, but were related to profession; farmers were at the highest risk of infection [4]. Significantly above-average prevalences were found among the Tujia and Miao ethnic minority [4]. According to the results of the second national survey, *F. buski* had considerably lost prominence as a human parasite [3]. With the exception of Zhejiang and Henan Provinces, a general decline in prevalence was noted (Table 11). However, pockets of high endemicity persist, e.g., in Hunan Province where all patients originated from Hanshou County where the local prevalence was 0.39%. Similarly, all infections detected in Shanghai originated from Chongming Island. 

**Table 11:** Endemic areas of fasciolopsiasis in China and change of prevalence between two national surveys.

Province	First National Survey			Second National Survey		
	No. Examined	No. Infected	Infection Rate (%)	No. Examined	No. Infected	Infection Rate (%)
Hubei	53,382	1,002	1.877	15,524	1	0.006
Shanghai	62,134	482	0.776	11,372	2	0.018
Hunan	63,794	210	0.329	15,233	6	0.039
Jiangsu	62,699	181	0.284	14,700	3	0.020
Jiangxi	52,069	100	0.192	20,154	30	0.149
Gansu	28,700	54	0.188	9,255	-	-
Guangxi	51,883	58	0.112	13,990	1	0.007
Anhui	54,392	60	0.110	14,873	6	0.040
Guangdong	61,517	60	0.098	17,014	-	-
Hainan	7,958	6	0.075	7,924	0	0
Zhejiang	55,284	40	0.069	15,863	17	0.107
Sichuan *	97,222	57	0.059	81,359	0	0
Fujian	53,416	29	0.053	20,195	+	+
Guizhou	52,938	5	0.094	15,958	-	-
Liaoning	51,405	4	0.008	22,767	-	-
Shandong	87,825	4	0.005	15,152	0	0
Henan	85,554	1	0.001	25,894	2	0.008

* including the number of participants from Chongqing which belonged to Sichuan Province during the first national survey and was an independent municipality during the second survey; - denoting the information is not known from available data; + denoting *F. buski* was detected from participants but no accurate figures can be obtained from the current data.

#### 3.8.3. Recent Advances in Research

Although human *F. buski* infections may be common in rural areas, few specific surveys have been conducted over the past decade. Only severe or ectopic infections, such as anemia and oedema, biliary fasciolopsiasis and intestinal obstruction, got attention from clinicians [167,168,169]. Detection methods such as serological testing have been developed as an alternative for faeces examination; ELISA with adult antigen of *F. buski* to detect specific antibodies in human sera showed high sensitivity and specificity, and a low rate of cross reactions with schistosomes and *Paragonimus* spp. [170]. Additionally, gastroscopy was proposed to diagnose early stage-fasciolopsiasis infections: in one study, 13 patients were diagnosed with *F. buski* infection by gastroscopy while their stool samples were egg-negative [171]. 

### 3.9. Clonorchiasis

#### 3.9.1. Parasites and Pathogenicity

Clonorchiasis is caused by a liver fluke, *Clonorchis sinensis*. The parasite is transmitted between snail, fish and mammals. Like *Fasciola* spp. aforementioned, adult worms of *C. sinensis* parasitize the biliary system of definitive hosts (human, cat, dog, *etc*.). The eggs are released with faeces to environment. Freshwater snail ingests the eggs and a few weeks later larvae develop into cercariae in the liver and are released from the snail body. Swimming cercariae invade fish and become metacercariae. The latter are infective for humans, dogs and cats.

The early stage of infection is normally asymptomatic, particularly in light infection. However, some patients can have symptoms associated with the inflammation in the liver and biliary ducts (Table 12). Chronic symptoms are mainly associated with dysfunction of biliary ducts and the gall bladder. Biliary obstruction can lead to jaundice. Bile stone or cholelithiasis is common outcome of chronic infection. *C. sinensis* also is one of the parasites that are confirmed as a cause of cancer; cholangiocarcinoma is the most significant a malignant outcome [172,173].

**Table 12:** Stages and Symptoms of Clonorchiasis.

Stages	Symptoms
Acute	✔ Fever ✔ Abdominal pain ✔ Diarrhea ✔ Rash ✔ Malaise ✔ Liver abscess ✔ Jaundice
Chronic	✔ Cholangitis ✔ Cholecystitis ✔ Cholelithiasis / bile stone ✔ Pancreatitis ✔ Cholangiocarcinoma

#### 3.9.2. Epidemiology

It is estimated that worldwide 15.3 million people were infected by *C. sinensis*, of which 84.3% are distributed in China [174]. According to the second national survey, the overall prevalence was 0.58% in the country [3]. In 27 endemic provinces the prevalence was as high as 2.4% and the number of infected people was 12.5 million [3]. The most heavily endemic provinces were Guangdong (17.48%), Guangxi (9.44%) and Heilongjiang (4.54%), which composed two hotspots in China, *i.e.*, southern and northeast centers. The prevalence among males (2.94%) was significantly higher than that among females (1.84%), which correlated with the differences in eating habits [3]. The group aged 50–59 year had the highest prevalence (9.16%). 

Clonorchiais is an increasing public health concern in public health in China [175]. Firstly, infected population is rapidly growing. The prevalence increased by 75% from the first national survey (0.33%) to the second national survey (0.58%). Secondly, infection with *C. sinensis* is the most common cause for surgery due to hepatobiliary and pancreatic diseases in endemic areas [176]. There might be over 1,000 patients admitted to a local hospital in endemic areas every year [177]. Third, cholangiocarcinoma is a malignant outcome of *C. sinensis* infection. Nearly 1,000 cases due to *C. sinensis* infection are diagnosed annually in China, which may still an underestimate [178,179].

#### 3.9.3. Recent Advances in Research

Bioinformatics is a hotspot in the field of *C. sinensis*-related research in recent years. An expression library for each stage of *C. sinensis* was established. Based on the libraries, individual genes and proteins of important function have been studied for diagnosis and drug targets. However, the conclusive findings have not been achieved. In 2011, the genome of *C. sinensis* has been published [180]. In-depth analysis will provide more insights for potential diagnosis and drug development.

Although most of infections may be asymptomatic, some can cause severe outcomes [175]. The large population with clonorchiasis imposes a huge disease burden on human health in China. To understand the burden, disability weight has been measured in a community-based study. The overall disability weights of the male and female were 0.101 and 0.050, respectively [181]. The overall disability weights of the age group of 5–14, 15–29, 30–44, 45–59 and 60+ were 0.022, 0.052, 0.072, 0.094 and 0.118, respectively [181]. The disease burden in the whole country should be estimated based on a clear epidemic profile. 

Praziquantel is confirmed to be efficacious against clonorchiasis [182]. However, adverse events are frequently observed after praziquantel administration [182]. Tribendimidine, artemether and artesuante showed high effectiveness in *C. sinensis*-infected rats and *in vitro* and hence might be potential new drug candidates for treating infections [183]. Recently, the Chinese anthelmintic drug tribendimidine was demonstrated to be highly effective against human infections [184].

### 3.10. Paragonimiasis

#### 3.10.1. Parasites and Pathogenicity

At least 29 species of *Paragonimus* have been described in China, among which 10 species are able to infect humans [4]. *P. westermani* and *P. skrjabini* (*Pagumogonimus skrjabini*) are the most common cause of human paragonimiasis. The life cycle of *Paragonimus* spp. involves three kinds of host. In mammalian definitive host, paired adult worms live in lung capsules. The eggs are released with sputum or swallowed and excreted in faeces. Hatched miracidiae penetrates the first intermediate host snail (*Melania* spp.), and ultimately develop into cercaria. Crustaceans (e.g., crabs and crayfish) serve as second intermediate hosts, which are infected via direct penetration of cercariae or ingestion of infected mollusks. Cercariae encyst and develop into metacercariae. Mammals acquire an infection by consuming raw or undercooked infected crustaceans. Metacercariae excyst in the small intestine and migrate through the intestinal wall to reach the abdominal cavity, enter the abdominal wall, and migrate to the pleural cavity.

Humans are permissive definitive hosts for several *Paragonimus* spp. and worms migrate along a mostly defined route to their final destination, the lung [185]. Therefore, the main symptoms are associated with lesions in the respiratory system. Common symptoms include cough, rusty sputum, *etc*. (Table 13) However, the central nervous system can also be involved if worms accidentally migrate through the soft tissues along the vessels of the neck and via the jugular foramen into the CNS [185]. Approximately 11.2–33.7% of *Paragonimus* spp. infections develop a cerebral involvement [3,186,187,188]. Cerebral paragonimiasis is potentially fatal [189].

**Table 13:** Stages and symptoms of Paragonimiasis.

Stages	Symptoms
Acute	✔ Abdominal pain ✔ Diarrhea ✔ Fever
Chronic	✔ Cough ✔ Rusty sputum
Infection outside respiratory system	✔ Symptoms related to eosinophilic meningitis ✔ Seizure ✔ Paralysis ✔ Subcutaneous migratory swellings

#### 3.10.2. Epidemiology

*Paragonimus* spp. was once widely distributed in China; 454 counties in 21 provinces were identified as endemic areas. Up to 1998, more than 23,000 human cases had been reported from 436 counties [3]. The main endemic area was localized at the middle reach of the Yangtze River. According to the second national survey, the serological prevalence of paragonimiasis was 1.71% (1,163/68,209) [3]. However, only 31 cases were parasitological diagnosed and accounted for 0.05%.

Paragonimiasis is a neglected parasitic disease. There is no specific control strategy for this disease up to date. Sporadic cases and even outbreaks were reported in recent years [190,191]. The symptoms due to *Paragonimus* infection resemble that of tuberculosis, a re-emerging infectious disease in China, and misdiagnosis is not uncommon. The absence of relevant knowledge in the clinical community is also challenging the control of paragonimiasis. 

#### 3.10.3. Recent Advances in Research

Traditionally, paragonimiasis is attributed to ingestion of raw or undercooked crabs. However, many patients deny the habit of eating raw or undercooked crabs. The probability of infection by drinking stream water contaminated with metacercariae is very low [192]. According to a recent report, cercariae successfully infected four dogs and one cat by feeding food with the parasite larvae [193]. This finding indicates that humans can possibly also be infected when drinking stream water contaminated with not metacercariae but cercariae. Of note, the density of cercariae can be much higher than that of metacercariae in stream water. 

Approximately 30 species of *Paragonimus* have been recorded in China. Recently, new species of *Paragonimus* as well as snail and crab hosts have been identified [194,195,196,197,198], indicating a complex phylogeny of the parasite and hosts. The validity of a species should be based on not only morphology but also molecular difference as well as ecological characteristics. However, molecular researches pertaining to the taxonomy of parasite and intermediate hosts are rare [199,200]. In addition, the worm specimens from patients are rarely identified by molecular tools. The exact number of species that can infect humans thus remains unknown. Therefore, the molecular tools should be applied to systematic studies regarding the phylogeny of *Paragonimus* as well as host animals in the near future, which in turn will help to understand the pathogenicity in humans.

### 3.11. Control Strategies for Water-Related Diseases

The majority of all human parasitic infections are diseases of poverty with a strong environmental component. Therefore, urbanization and socioeconomic development which have improved the living conditions of many Chinese have contributed to decrease of the prevalence of parasitic diseases so that they are no longer major public health problems. However, the burden due to parasitic diseases is still considerable in certain areas and populations across China, owing to the inequality in economic development, public services including health care and preventive medicine, and environmental conditions. Of note, many policies conceived for the economic development of the country, particularly those aimed at the countryside and relatively poor regions, significantly impact the epidemiology of parasitic diseases; even if they were not conceived to directly control them. In addition, numerous targeted measures for diseases control mentioned below are being implemented.

#### 3.11.1. Legal Framework

The “Law of the People’s Republic of China on the Prevention and Treatment of Infectious Diseases” was issued in 1989 and amended in 2004. Aimed at the control of infectious diseases, it mandates that medical personnel with knowledge about individuals with any of the six designated parasitic diseases, namely amoebiasis dysentery, schistosomiasis, malaria, leishmaniasis, echinococcosis and filariasis, are obliged to report all cases via the national reporting system. The responsible national-level public health departments then respond to these reports and initiate appropriate steps and interventions. The reporting system has greatly facilitated the identification of outbreaks and monitoring of trends in prevalence and incidence. Furthermore, the “Schistosomiasis Prevention and Control Regulation” was issued in 2006, mandating a coordinated effort by several departments of the central and local governments to guarantee the full implementation of the comprehensive control strategy [2]. 

#### 3.11.2. Water Supply and Sanitation

Awareness and concern for public health are rapidly increasing in China. A reliable supply of clean drinking water in adequate amounts is now widely demanded as a basic service, albeit still not available to millions of inhabitants. The new “National Drinking Water Criteria (GB5749-2006)” were published in 2006 and list *Cryptosporidium* spp. and *G.* *intestinalis* as indicators for water quality. The current limit to consider drinking water as safe for human consumption is a concentration of less than one *G. intestinalis* cyst or *Cryptosporidium* oocyst per 10 L water. Standard examination methods for water samples are also provided. In urban areas, utilities have taken the new criteria into account. The possibility of schistosomiasis japonica transmission and the expansion of the endemic area have become elements of environmental and health impact assessments of water resource management projects such as the South-North Water Transfer Project and the Three Gorges Dam [201,202,203].

Sanitation and the proper management of wastewater are important elements for ensuring the supply of clean drinking water. Improving sanitation includes the construction of latrines or water-flush toilets and sewers, the collection of wastewater of domestic and industrial origin and its separation from environmental water bodies, and waste water treatment. Almost all studies on the effect of latrine construction and usage demonstrate their effectiveness in reducing the prevalence of different water-based and -related infectious diseases [204,205]. However, latrine construction is seldom considered as an independent control tool but implemented in the frame of general development and urban or rural rehabilitation programs. There are currently no national laws or criteria for faeces management in China. Still, more and more sewage treatment plants are constructed based on local regulations and initiative, especially in urban areas. In rural areas, septic tanks and biogas facilities at household or community level are promoted. They not only provide gas as a substitute fuel but also sterilize the sludge, including parasite eggs [204].

#### 3.11.3. Innovative Approaches for the Control of Water-Related Parasitic Infections

Diseases occur in a complex socio-ecological context characterized by feedback loops across space and time, self-organization, holarchies, and sudden changes in organization when thresholds are reached [206]. Therefore, single-minded control programs usually fail to effectively control the target disease. A sustainable control strategy must incorporate multiple scales and perspectives, and high degrees of uncertainty [206]. The comprehensive strategy for the control of schistosomiasis japonica that is promoted in China illustrates this ecosystem-based approach [132]. The measures include replacing labor animals with machines, prohibiting the grazing of cattle in grasslands adjacent to water bodies, supplying tap water, constructing latrines and the promotion of biogas pools. Community-based health education and synchronous praziquantel-based chemotherapy for all villagers and their cattle are implemented in order to rapidly decrease the prevalence. The new control strategy eschews the use of chemical molluscicides, which have proven to result in environmental pollution and damage [207]. 

## 4. Conclusions

Water-related parasitic diseases have generally declined in China. This has often been attributed to socioeconomic development, which has improved living conditions and alleviated the financial burden due to parasitic diseases in many areas. However, some parasitic diseases still occur at considerable prevalence in certain areas and populations, owing to the heterogeneity in natural conditions and economic development and the inequity of access to public services including preventive and curative health care. Emerging and re-emerging pathogens and risk factors are also challenging the achievements made with regard to the control of parasitic diseases [208]. The growing population of immunocompromised individuals due to HIV, chronic diseases, cancer and immunosuppressive drugs is vulnerable to opportunistic parasite infections, such as cryptosporidiosis and cyclosporiasis.

The majority of all parasitic infections are diseases of poverty [1]. However, some parasitic diseases, especially helminthiases, occur in a complicated context and call for ecosystem-based approaches. Controlling major sources of infection including humans and animals, changing land-use patterns, and adapting to climate change are important components of ecosystem-based inventions. 
